# Role of microRNA-92a in metastasis of osteosarcoma cells in vivo and in vitro by inhibiting expression of TCF21 with the transmission of bone marrow derived mesenchymal stem cells

**DOI:** 10.1186/s12935-019-0741-1

**Published:** 2019-02-12

**Authors:** Shuai Cao, Liangde Jiang, Lulu Shen, Zhizheng Xiong

**Affiliations:** 1 Department of Orthopedics, Renhe Hospital, Beijing, 102600 People’s Republic of China; 2 Department of Orthopedics, Yueyang Second People’s Hospital, Yueyang, 414000 People’s Republic of China

**Keywords:** Osteosarcoma, MicroRNA-92a, TCF21, Invasion, Migration, Proliferation

## Abstract

**Background:**

This study aims to investigate the role of microRNA-92a (miR-92a) in metastasis of osteosarcoma (OS) cells in vivo and in vitro by regulatingTCF21 with the transmission of bone marrow derived mesenchymal stem cells (BMSCs).

**Methods:**

BMSCs were isolated, purified and cultured from healthy adult bone marrow tissues. The successfully identified BMSCs were co-cultured with OS cells, and the effects of BMSCs on the growth metastasis of OS cells in vitro and in vivo were determined. qRT-PCR and western blot analysis was used to detect the expression of miR-92a and TCF21 in OS cells and OS cells co-cultured with BMSCs. Proliferation, invasion and migration of OS cells transfected with miR-92a mimics and miR-92a inhibitors was determined, and the tumorigenesis and metastasis of OS cells in nude mice were observed. Expression of miR-92a and TCF21 mRNA in tissue specimens as well as the relationship between the expression of miR-92a and the clinicopathological features of OS was analyzed.

**Results:**

BMSCs promoted proliferation, invasion and migration of OS cells in vitro together with promoted the growth and metastasis of OS cells in vivo. Besides, high expression of miR-92a was found in OS cells co-cultured with BMSCs. Meanwhile, overexpression of miR-92a promoted proliferation, invasion and migration of OS cells in vitro as well as promoted growth and metastasis of OS cells in vivo. The expression of miR-92a increased significantly, and the expression of TCF21 mRNA and protein decreased significantly in OS tissues. Expression of miR-92a was related to Ennecking staging and distant metastasis in OS patients.

**Conclusion:**

Collectively, this study demonstrates that the expression of miR-92a is high in OS and BMSCs transfers miR-92a to inhibit TCF21 and promotes growth and metastasis of OS in vitro and in vivo.

## Background

Osteosarcoma (OS), is the most commonly occurring primary malignant bone tumor, which mainly affects the metaphysis of long bones amongst children and adolescents [1]. OS comprises approximately 2.4% of all malignancies in pediatric patients, and about 20% of all primary bone cancers [1, 2]. It is reported that there are some common risk factors contribute to OS development, including alkylating agents, ionising radiation, hereditary retinoblastoma, Paget’s disease, the Li-Fraumeni familial cancer syndrome as well as other chromosomal abnormalities [3]. Despite many tumors respond to chemotherapy initially, OS patients with relapsed disease and metastatic disease continue to show extremely poor survival outcomes [4, 5]. Bone marrow-derived mesenchymal stem cells (BMSCs) are described as locally adjacent to the tumor tissues, which might directly interact with tumor cells [6]. Besides, BMSCs is able to promote the proliferation and invasion of OS cells, which may be involved in the SDF-1/CXCR4 axis [7]. Nevertheless, the mechanisms for initiation and progression of OS are not clear. Recently, it has been reported that the expression and function of specific microRNA (miRNA) in OS will potentially result in the identification of new biomarkers and therapeutic targets in OS.

MiRNAs play significant roles in several cellular processes, including proliferation, apoptosis, differentiation and development by simultaneously modulating the expression of hundreds of genes [8, 9]. Indeed, specific miRNAs is demonstrated to contribute to tumor growth, metastasis and drug resistance in OS [10–13]. Among which, miR-199a and miR-34a has been suggested to repress the cell growth and migration in cancers [14, 15]. MiR-92a family exerts it function in regulating both the development of mammalian organs such as heart, lungs and immune system and the formation of blood vessels [16]. Additionally, miR-92a acts as a biomarker and drug-target in OS, and it also exerts an oncogenic role via targeting PTEN/AKT pathway [17]. Besides, miR-92a probably acts as a driver of tumor progression through targeting FBXW7, which highlighting the potential functions of miR-92a on prognosis and treatment of OS [18]. Transcription factor 21 (TCF21) belongs to the basic helix-loop-helix transcription factor family, which plays a vital role in modulating cell development and differentiation [19, 20]. Except that, TCF21 gene polymorphism has been demonstrated to be related to the risk and outcome of OS in an east Chinese population [21]. Based on the aforementioned evidence, we assure that BMSCs transfers miR-92a to inhibit TCF21 and promotes growth and metastasis of OS in vitro and in vivo.

## Materials and methods

### Isolation and culture of human BMSCs

Bone marrow specimens from healthy adult donors were collected and approved by the ethics committee of Renhe Hospital before the collection, and informed consent was signed by all the participates. The collected bone marrow tissues were added with 10 mL DMEM (Gibco, Grand Island, NY, USA), mixed, centrifuged with the supernatant discarded. After washing with heavy cell suspension, 4 mL D-Hanks solution (Invitrogen, Carlsbad, CA, USA) and 4 mL cell isolate solution (0.56: 0.44) were added. Monocyte layer was collected after centrifugation and washed with DMEM medium containing 10% fetal bovine serum (FBS, Gibco, Grand Island, NY, USA). BMSCs were inoculated into a 25 mL culture flask (5 × 10^5^ cells/cm^2^) and cultured in an incubator of 5% CO_2_ at 37 °C for 3 days and then changed solution every 2 days. After the cells reached about 70–80% confluency, the cells were detached and cultured with 2.5 g/L trypsin. Cell morphology was observed under an inverted phase contrast microscope.

### Identification of surface markers of human BMSCs

The cells at passage 3 with good growth state were detached and then prepared for single cell suspension with phosphate buffer saline (PBS) containing 1% bovine serum albumin (BSA). About 1 × 10^6^ cells/l00 μL were packed into several Eppendorf (EP) tubes, the mesenchymal stem cells that were packed into each EP tube were added with fluorescent-labeled antibody: FITC-CD45, PE-CD29, FITC-CD90, PE-CD34, PE-CD105 and isotype control (Abcam Cambridge, MA, USA), respectively. The cells were dissolved on ice for 1 h, then washed with PBS containing 1% BSA for 3 times, and then suspended again with PBS. Make sure that there were at least 1 × 10^5^ cells for detection per tube. A flow cytometer was used for detection (Becton, Dickinson and Company, Franklin lake, New Jersey, USA).

### Identification of osteogenic and adipogenic differentiation ability of human BMSCs

The cells at passage 3 were prepared into single cell suspensions and inoculated with 3 × 10^4^ cells/well in a 6-well plate and cultured in 2 mL complete culture medium (Gibco, Grand Island, NY, USA) for each well. When the cells reached 80% confluency, the culture medium was abandoned, and 2 mL osteogenic induced differentiation complete medium (Cyagen Biosciences Inc., Guangzhou, China) was added for culture. The solution was completely changed every 72 h. After 3 weeks of induction, the cells were fixed with 4% paraformaldehyde, stained with 0.1% alizarin red S (Cyagen Biosciences, Santa Clara, CA, USA) for 20 min, and the formation of intracellular calcium nodules was observed under a microscope.

The cells at passage 3 were prepared into single cell suspensions and inoculated with 2 × 10^4^ cells/well in a 6-well plate and cultured in 2 mL complete culture medium for each well. The solution was completely changed every 72 h. When the cells reached 100% confluency, the culture medium was abandoned, and 2 mL adipogenic differentiation complete medium A solution (Cyagen Biosciences Inc., Guangzhou, China) was added for culture. After 72 h, A solution was abandoned and 2 mL adipogenic differentiation culture medium B solution was added for culturing for 24 h, and then B solution was abandoned and 2 mL A solution was added for culture. After 3 cycles, the cells were cultured in B solution for 2 days, then fixed with 4% polycarboxylic acid for 20 min, stained with 2% oil red O (Cyagen Biosciences Inc., Guangzhou, China) for 30 min and washed with 70% ethanol. The lipid droplet formation was observed under a microscope.

### Co-culture of human BMSCs and OS cells

OS cell lines 143B and SaOS2 were selected for experiment. The cells were cultured with BME medium containing 10% FBS, 100 unit/mL penicillin and 100 μg/mL streptomycin sulfate (Gibco, Grand Island, NY, USA) in a 5% CO_2_ incubator at 37 °C. The solution was changed every 3 days. When the cell density reached 80–90%, the passage culture began.

BMSCs at passage 5 were co-cultured with 143B or SaOS2 cells in the upper and lower chambers of Transwell in the 6-well plate. The density of the cells in the upper chamber and the lower chamber was 20,000 and 30,000, respectively. After 72 h, 80% cell density was observed. After washing with PBS, BMSCs-143B and BMSCs-SaOS2 cells were selected for subsequent experiments. Uncocultured 143B and SaOS 2 cells were used as controls.

### MTT assay

The cell suspensions were diluted at a certain concentration and then inoculated into a 96-well plate at the density of 5 × 10^4^ per well. Each group was divided into 6 parallel wells. After the cells reached 80% confluency, the cells were treated in groups according to the experiment grouping mentioned above. The cells were cultured for 24 h, 48 h, 72 h and 96 h, respectively, and 20 μL MTT solution (Sigma Aldrich (St. Louis, MO, USA)) was added and incubated in a 37 °C incubator for 4 h. The MTT solution was abandoned and the dimethyl sulfoxide (DMSO, 150 μL, Sigma Aldrich (St. Louis, MO, USA)) was added into each well. After shaking for 10 min, the optical density (OD) value of each well was measured at the wavelength of 490 nm. The experiment was repeated three times.

### Transwell assay

After transfection of 48 h, cells were detached in each group. Each Transwell chamber coated with matrigel (1:8, 80 μL) was inoculated with 1 × 10^5^ cells, with 100 μL serum-free DMEM supplemented. The basolateral chamber was added with complete medium. After 24 h of incubation, cells of the apical chamber were wiped by a cotton swab. Then cells were fixed in 4% paraformaldehyde for 15 min and stained by crystal violet for 10 min. Under a microscope, 5 fields of view were randomly selected for photographing and counting. The number of cells adhering to the matrigel of the side in the basolateral chamber was considered as the number of invasive cells. The experiment was conducted in triplicates.

### Scratch test

At the back of the 6-well plate, the marker pen was used to draw a uniform horizontal line against the ruler about every 0.8 cm or so, crossing the well. Each well passed through at least 5 lines. When about 5 × 10^5^ cells were added into each well, the cells reached 100% confluency. The next day, a 10 μL gun head was perpendicular to the back of the horizontal line against the ruler scratch, and the gun head was vertical and could not be tilted. After scratching, the cells were gently washed with PBS for 3 times, then gently adhered to the wall and added with PBS. The cells were washed and removed, then added to the culture medium and cultured in a CO_2_ incubator at 37 °C. At the time point of 0 h and 24 h, the sampling was performed so as to take pictures under an inverted microscope. The wound healing area was calculated with National instrument Vision Assistant 8.6 software. Cell migration rate = wound healing area/initial scratch wound area × 100%. The experiment was repeated three times.

### Quantitative reverse transcription polymerase chain reaction (qRT-PCR)

The one-step method of Trizol (Invitrogen, Carlsbad, CA, USA) was used to extract the total RNA in cells and tissues, and the high-quality RNA was confirmed by ultraviolet (UV) analysis and the detection of formaldehyde denaturation electrophoresis. The complementary DNA (cDNA) was obtained by avian myeloblastosis virus (AMV) reverse transcriptase after the acquirement of l μg RNA. PCR primer was designed and synthesized by Invitrogen, Carlsbad, CA, USA (Table 1). U6 and glyceraldehyde phosphate dehydrogenase (GAPDH) was used as internal controls. The PCR amplification conditions were: pre-denaturation at 94 °C for 5 min, a total of 40 cycles of denaturation at 94 °C for 40 s, annealing at 60 °C for 1 min, extension at 72 °C for 1 min and finally, extension at 72 °C for 10 min. The product was verified by agarose gel electrophoresis. The threshold cycle (Ct) value of each reaction tube was obtained by manually selecting the threshold at the lowest point of parallel rise of each logarithmic expansion curve. 2^−ΔΔCt^ method was used to analyze the ratio relation of target gene expression between the experimental group and the control group. The formula is as follows:

$$ \Delta \Delta {\text{Ct }} = [{\text{Ct}}_{{\left( {{\text{target}}\,{\text{gene}}} \right)}}  - {\text{ Ct}}_{{\left( {{\text{internal control gene}}} \right)}} ]\,_{{{\text{experimental group}}}}  - {\text{ }}\left[ {{\text{Ct}}_{{\left( {{\text{target}}\,{\text{gene}}} \right)}}  - {\text{ Ct}}_{{\left( {{\text{internal control gene}}} \right)}} } \right]\,_{{{\text{control group}}}}. $$

The experiment was repeated for three times.

**Table 1:** The primer sequence

Gene	Sequence
miR-92a	F: 5′- GCTGAGTATTGCACTTGTCCCG -3′
	R: 5′- GTGTCGTGGAGTCGGCAA -3′
U6	F: 5′-CTCGCTTCGGCAGCACA -3′
	R: 5′- AACGCTTCACGAATTTGCGT -3′
TCF21	F: 5′- CCAGCTACATCGCCCACTTG -3′
	R: 5′- CTTTCAGGTCACTCTCGGGTTTC -3′
GAPDH	F: 5′- TGGGTGTGAACCATGAGAAG -3′
	R: 5′- GTGTCGCTGTTGAAGTCAGA -3′

*F* forward, *R* reverse, *miR*-*92a* microRNA-92a, *GAPDH* glyceraldehyde phosphate dehydrogenase

### Western blot analysis

The proteins from cells in each group were extracted and the protein concentrations were determined according to the instructions of the bicinchoninic acid (BCA) assay (Wuhan Boster Biological Technology LT, Wuhan, China). The extracted protein was added to the sample buffer and then boiled at 95 °C for 10 min, with each well loading for 30 μg protein. Following separation of 10% sodium dodecyl sulfate polyacrylamide gel electrophoresis (SDS-PAGE) (Wuhan Boster Biological Technology LT, Wuhan, China), protein samples were transferred to a nitrocellulose (NC) membrane using the wet transfer method, with the electrophoretic voltage from 80 v to 120 v, the trarsmembrane voltage of 100 mv for 45–70 min. Subsequently, the protein samples were transferred to polyvinylidene fluoride (PVDF) membrane and blocked with 5% BSA for 1 h. Afterwards, the membranes were added with the primary antibodies of TCF21 (1:1000) and β-actin (1:3000) (Abcam, Cambridge, MA, USA) and incubated at 4 °C overnight. The membranes were rinsed with Tris-buffered saline and Tween 20 (TBST) for 3 times, each time for 5 min. The corresponding secondary antibodies were incubated at room temperature for 1 h to wash the membranes for 3 times, each time for 5 min. An electrogenerated chemiluminescence (ECL) solution was used for developing. β-actin was regarded as an internal control. Bio-rad Gel Dol EZ formatter (GEL DOC EZ IMAGER, Bio-rad, California, USA) was used for developing. The gray value analysis of target band was analyzed by Image J software. The experiment was repeated for three times.

### In situ tumor of tibia model in nude mice

The healthy Specific pathogen Free (SPF) female BALB/C nude mice, aged 4–6 weeks old and weighted (18 ± 2) g, were purchased from Beijing Vital River Laboratory Animal Technology Co., Ltd. (Beijing, China). The nude mice were raised in a pathogen-free environment in the laboratory of immunodeficient animals in Renhe Hospital. Animal experiments were approved by the Ethical Committee of Laboratory Animals in Renhe Hospital.

The 143B cells that had a higher tendency of spontaneous lung metastasis were selected for in vivo metastasis study. After each 143B cell and luciferase reporter gene was grown close to confluence, the cell density was adjusted to 2 × 10^7^ cells/mL by suspension of aseptic PBS. After the nude mice were anesthetized and treated, each 143B cell was injected into the outer bone of the lateral tibia of the nude mice, and 50 μL (containing 1 × 10^6^ cells) of the cell suspension was injected at each site, and 6 nude mice were injected into each cell. The nude mice were intraperitoneally injected with 200 μL 150 mg/kg D-fluorescein (Promega, Madison, Wisconsin, USA). After 10 min, the photons from luciferase bioluminescence were counted according to the instructions of the IVIS imaging system (Perkin Elmer, Waltham, California, USA). Bioluminescence imaging was used to measure the size of tumor in situ of nude mice every 3 days after the tumor appeared. The development of lung metastasis in OS was monitored at 1st, 3rd and 5th week. After 5 weeks, the nude mice were killed, the tumor was weighed, and the lung tissues around the nodules were collected and fixed with 10% formalin solution. After hematoxylin–eosin (HE) staining, the lung metastasis was observed under a microscope.

### Cell grouping

The 143B or SaOS2 cells in good growth state were assigned into 5 groups: blank group, mimics negative control (NC, cells transfected with mimics NC), inhibitors NC (cells transfected with inhibitors NC), miR-92a mimics (cells transfected with miR-92a mimics) and miR-92a inhibitors (cells transfected with miR-92a inhibitors). mimics NC, miR-92a mimics, inhibitors NC and miR-92a inhibitors were purchased from (Shanghai GenePhama Co., Ltd., Shanghai, China. The cells were transfected with the instructions of Lipofectamine™ 2000 (Invitrogen, Carlsbad, CA, USA). After 48 h, the cells were collected and the expression of miR-92a and TCF21 were detected. MTT assay was used to detect cell proliferation. Transwell assay was used to detect cell invasion ability, scratch test to detect cell migration ability, as well as in situ tumor test in nude mice to detect tumor growth and metastasis in vivo.

### Dual luciferase reporter gene assay

Bioinformatics software http://www.targetscan.org was used to predict the targeting relationship between miR-92a and TCF21 and the binding sites of miR-92a to TCF21 3′UTR. TCF21 3′UTR promoter sequence containing miR-92a binding site was synthesized and TCF21 3′UTR wild type plasmid (TCF21-WT) was constructed. On the basis of this plasmid, the mutant type plasmid TCF21 3′UTR (TCF21-MUT) was constructed. The procedure was carried out according to the instructions of plasmid extraction kit (Promega, Madison, Wisconsin, USA). The cells in the logarithmic growth were inoculated into the 96-well plate and transfected with Lipofectamine 2000 when the cell density was about 70%. TCF21-WT and TCF21-MUT were mixed with mimics NC and miR-92a mimics (Shanghai GenePharma Co., Ltd (Shanghai, China)) respectively, and then co-transfected into 293T cells. After 48 h of transfection, luciferase activity was collected and lysed. The luciferase activity was detected by a Glomax20/20 luminometer (Promega, Madison, Wisconsin, USA) with a luciferase detection kit (BioVision, San Francisco, CA, USA). The experiment was repeated three times.

### Tissue specimen

The specimens of 58 patients with OS diagnosed and treated in Renhe Hospital from January 2016 to March 2018 were selected. The patients were enrolled if they met the following criteria: patients were confirmed with OS by pathological examination after operation; patients didn’t receive preoperative radiotherapy, chemotherapy and other clinical adjuvant therapy. The patients were excluded if they were complicated with severe systemic diseases such as heart, liver and kidney, as well as other malignant tumors. The clinical data of all patients were collected in detail. Thirty osteochondroma (benign bone lesions) tissue specimens from the Pathology Department of Renhe Hospital during the same period were selected as controls. The study was approved by the Ethics Committee of Renhe Hospital, and all the participates signed the informed consent.

### In situ hybridization (ISH)

The paraffin sections of pathological specimens were dewaxed with xylene, washed in water, and digested in a mixture of 0.8% pepsin and hydrochloric acid for 10 min (exposed to RNA fragments) at 37 °C. The sections were dehydrated with ethanol, denatured at 98 °C for 10 min, and prehybridized at 37 °C for 1 h. After removing the pre-hybridization solution, the sections were added with the hybridization solution containing miR-92a or TCF21 probe (Guangzhou Ruibo Biotechnology Co., Ltd., Guangzhou, China) for overnight hybridization at 40 °C. The digoxin antibody labeled with alkaline phosphatase was added for incubation at room temperature for 30 min, washed twice according to the above method, then added with BCIP/NBT and stained with 0.3% hematoxylin in the dark, followed by ethanol dehydration, xylene clearance, and sealing.

According to the double-blind observation by two pathologists, the positive signals of miR-92a were mainly located in the cytoplasm and interpreted according to the cytoplasmic pornography. Positive score was divided into four grades: no expression or background color was the same, and the percentage of staining less than 20% was negative (−); Light brown or yellowish, 20–50% of the staining percentage was weakly positive (+); Brown-yellow and 50–70% of the staining percentage was moderately positive (++); Brown and the percentage of staining more than 75% was strong positive (+++). (+), (++) and (+++) were considered as positive expression.

### Immunohistochemistry

The paraffin sections were dewaxed and incubated at room temperature for 5–10 min with 3% hydrogen peroxide. After several washes, the sections were blocked with 10% normal goat serum t and incubated at room temperature for 10 min. Next, the sections were supplemented with TCF21 primary antibody (Abcam, Cambridge, MA, USA) and kept in a refrigerator at 4 °C for overnight incubation. After adding with the secondary antibody, the sections were incubated in a 37 °C incubator for 30 min. After diaminobenzidine (DAB) coloration, the sections were re-stained with hematoxylin, dehydrated, cleared and sealed.

The results of double-blind observation by two pathologists showed that the positive signals of TCF21 were located in the nucleus or cytoplasm, mainly in the cytoplasm. According to the degree of histological section, 0 point for non-staining, 1 point for light yellow, 2 points for brown yellow and 3 points for brown. According to the percentage of positive cells in visual field cells, the score was 0 point in 0–5%, 1 point in 6–25%, 2 points in 26–50%, 3 points in 51–75% and 4 points in > 75%. The product score of the above two items in each slice was 0 point for negative (−), 1–4 points for weak positive (+), 5–8 points for moderate positive (++), and 9–12 points for strong positive (+++). (+), (++) and (+++) were considered as positive expression.

### Statistical analysis

All the data were analyzed by SPSS 21.0 software (SPSS, Inc, Chicago, IL, USA). The measurement data were expressed as mean ± standard deviation. The t test was used for the comparison between the two groups, and one-way analysis of variance (ANOVA) was used for the comparisons among multiple groups. After ANOVA analysis, the Fisher’s least significant difference t test (LSD-t) was used for pairwise comparisons. All tests were 2-sided and *P* values ≤ 0.05 were considered statistically significant.

## Results

### Identification of BMSCs

**Fig. 1:**
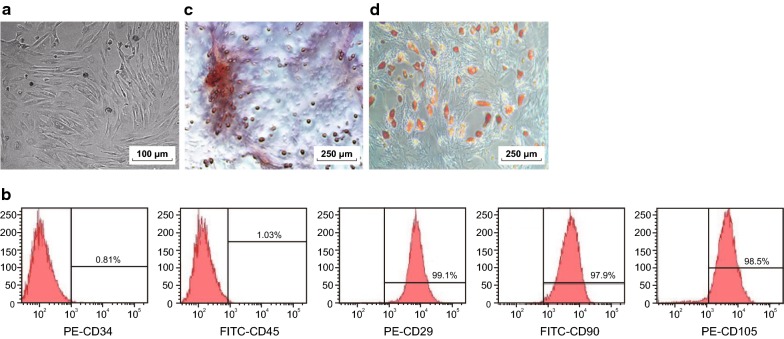
Identification of BMSCs. **a** Observation of cell morphology by an inverted microscope (×100); **b** identification of cell surface markers by flow cytometry; **c** identification of osteogenic differentiation ability of cells by alizarin red staining (×40); **d** identification of adipogenic differentiation ability of cells by oil red O staining (×40). The experiment was repeated three times

The observation of BMSCs under an inverted microscope showed that the BMSCs cultured in vitro were homogeneous in distribution and uniform in morphology. The cells were detached and subcultured to the passage 3. The cells were spindle-shaped average growth and typical whirlpool (Fig. 1a).

Identification of surface markers of human BMSCs was determined by flow cytometry. The surface markers (CD29 [99.10 ± 0.45]%, CD90 [97.90 ± 0.42]% and CD105 [98.50 ± 0.37]% with high expression and CD34 [0.81 ± 0.07]% and CD45 [1.03 ± 0.10]% with low expression of mesenchymal stem cells in BMSCs cultured in vitro was identified to meet with the immunophenotypic characteristics of BMSCs (Fig. 1b).

After osteogenic induction of human BMSCs, a large number of red staining of calcium nodules could be seen under a microscope by alizarin red staining, which showed that the cells had good osteogenic differentiation function (Fig. 1c). After the intervention of fat inducer, the red dye granules were appeared in the cytoplasm of the cells, and a large number of lipid droplets could be seen at the same time under a microscope by oil red O staining, indicating the existence of adipose differentiation function of the cells (Fig. 1d). The results indicated that human BMSCs was isolated and cultured successfully.

### BMSCs promote proliferation of OS cells in vitro

**Fig. 2:**
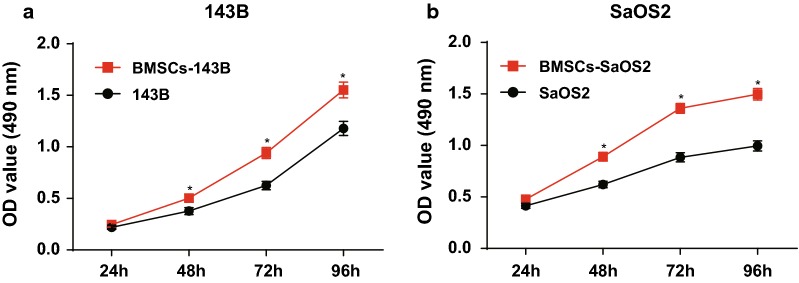
BMSCs promote proliferation of OS cells in vitro. Repetitions = 3; the data was analyzed by the t test; *, *P *< 0.05 vs. 143B cells or SaOS2 cells

Proliferation of 143B and SaOS2 cells after co-cultured with BMSCs was measured by MTT assay. The results suggested that the proliferation rates of BMSCs-143B and BMSCs-SaOS2 cells were increased significantly after 48 h compared with those in un-cocultured 143B and SaOS2 cells (all *P *< 0.05; Fig. 2), indicating that BMSCs promote proliferation of OS cells in vitro.

### BMSCs promote invasion and migration of OS cells in vitro

**Fig. 3:**
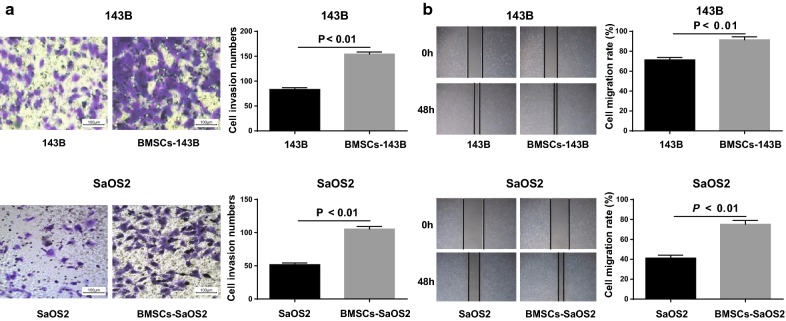
BMSCs promote the invasion and migration of OS cells in vitro. **a** Transwell assay detection of the number of cell invasion in each group (× 100); **b** detection of cell migration in each group by scratch test. Repetitions = 3; the data was analyzed by the t test

The invasion ability of 143B and SaOS2 cells was detected by Transwell assay before and after co-cultured with BMSCs. The results showed that the number of cell invasion of 143B and SaOS2 cells were increased significantly after co-cultured with BMSCs compared with un-cocultured 143B and SaOS2 cells (all *P* < 0.05; Fig. 3a).

The migration ability of 143B and SaOS2 cells was detected by scratch test before and after co-cultured with BMSCs. The results showed that the migration ability of BMSCs-143B and BMSCs-SaOS2 cells was significantly higher than that in un-cocultured 143B and SaOS2 cells (all *P* < 0.05; Fig. 3b). These results suggest that BMSCs can promote the invasion and migration of OS cells in vitro.

### BMSCs promote the growth and metastasis of OS cells in vivo

**Fig. 4:**
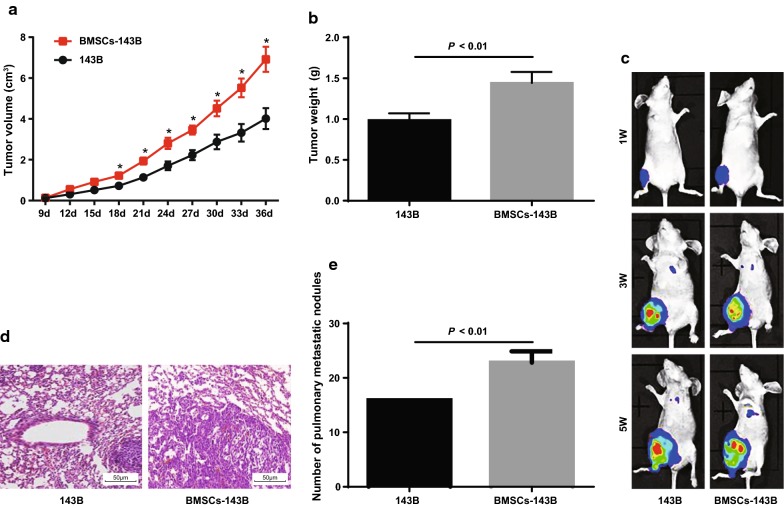
BMSCs promote the growth and metastasis of OS cells in vivo. **a** Tumor growth curve of two groups of nude mice; **b** tumor weight in two groups of nude mice; **c** lung metastasis in two groups of nude mice; **d** observation of lung metastasis in two groups of nude mice by HE staining (× 200); **e** number of lung metastatic nodules in two groups of nude mice; N = 6; the data was analyzed by the t test; *, *P *< 0.05 vs. 143B cells

Effects of BMSCs on in vivo growth and metastasis of 143B cells in nude mice was using in situ implantation model of tibia. The results showed that compared with 143B cells, the growth rate of BMSCs-143B cells was significantly increased, tumor volume and tumor weight were also significantly increased (all *P* < 0.05; Fig. 4a, b).

At 1 week after cell injection, the signals from luciferase bioluminescence were detected in the primary injury sites of 143B cells and BMSCs-143B cells, but no signals were detected in the lungs. At the third week, 1 of the 6 mice injected with 143B cells showed the signal in the lung region, and 3 of the 6 mice injected with BMSCs-143B cells showed the signal in the lung region with metastatic lesions. On the 25th day and the 31st day, one nude mouse died in BMSCs-143B cells; on the 28th day, one nude mouse died in 143B cells; the dead nude mice were confirmed by autopsy to have lung metastases. Lung metastasis was detected in both surviving nude mice after 31 days. Compared with 143B cells, the number of lung metastatic nodules in BMSCs-143B cells increased significantly (*P *< 0.05; Fig. 4c–e). These results suggest that BMSCs can promote the growth and metastasis of OS cells in vivo.

### High expression of miR-92a in OS cells co-cultured with BMSCs

**Fig. 5:**
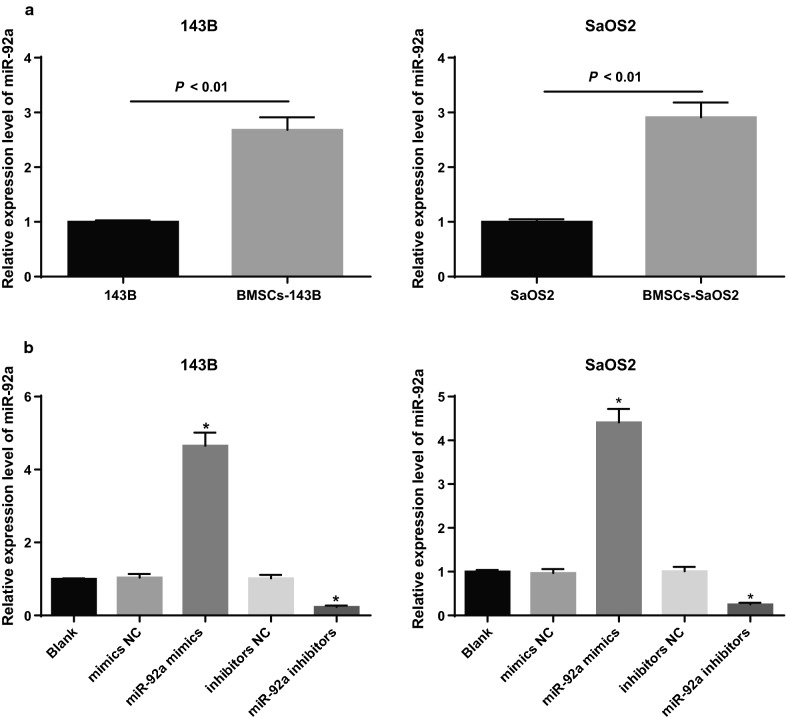
High expression of miR-92a in OS cells co-cultured with BMSCs. **a** Detection of miR-92a expression in 143B and SaOS2 cells before and after co-culture by qRT-PCR; **b** expression of miR-92a in 143B and SaOS2 cells detected by qRT-PCR; Repetitions = 3; the data was analyzed by the t test or one-way ANOVA; After ANOVA analysis, the LSD-t was used for pairwise comparison; *P < 0.05 vs. the blank group

The expression of miR-92a in 143B and SaOS2 cells as well as BMSCs-143B and BMSCs-SaOS2 cells were detected by qRT-PCR. The results revealed that the expression of miR-92a was significantly higher in the cell lines of BMSCs-143B and BMSCs-SaOS2 than in the 143B and SaOS2 cells (all *P* < 0.05; Fig. 5a). These results suggest that the functions of BMSCs on OS cells may be related to miR-92a.

The expression of miR-92a in 143B and SaOS2 cells in each group was detected by qRT-PCR. The results indicated that the expression of miR-92a in the miR-92a mimics group was significantly higher but the expression of miR-92a in the miR-92a inhibitors group was significantly lower than that in the blank and mimics NC groups (all *P *< 0.05). There was no significant difference in the expression of miR-92a among the mimics group, inhibitors NC group and the blank group (all *P* > 0.05; Fig. 5b). The miR-92a mimics and miR-92a inhibitors was successfully intervened.

### Overexpression of miR-92a promotes proliferation of OS cells in vitro

**Fig. 6:**
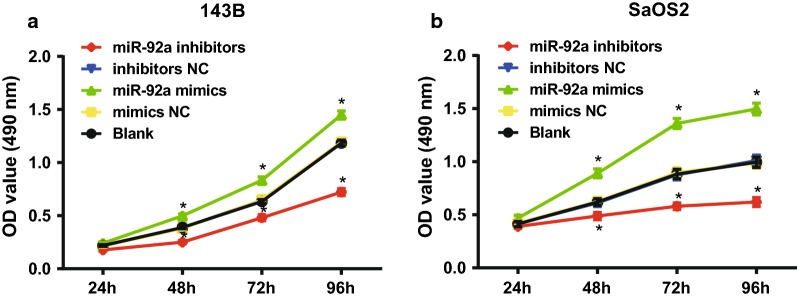
Overexpression of miR-92a promotes proliferation of OS cells in vitro. Repetitions = 3; the data was analyzed by the t test or one-way ANOVA; After ANOVA analysis, the LSD-t was used for pairwise comparison; *P < 0.05 vs. the blank group

The proliferation of 143B and SaOS2 cells in each group was detected by MTT assay. The results suggested that there was no significant difference in cell proliferation rate and proliferation activity at each time point among the blank, mimics NC and inhibitors NC groups (all *P* > 0.05). Compared with the blank and mimics NC groups, the proliferation rate and proliferation activity were significantly increased after 48 h of cells in the miR-92a mimics group (all *P *< 0 05). Relative to the blank and inhibitors NC group, the proliferation rate and proliferation activity of cells in the miR-92a inhibitors group was significantly decreased after 48 h (all *P* < 0.05; Fig. 6).

### Overexpression of miR-92a promotes invasion and migration of OS cells in vitro

**Fig. 7:**
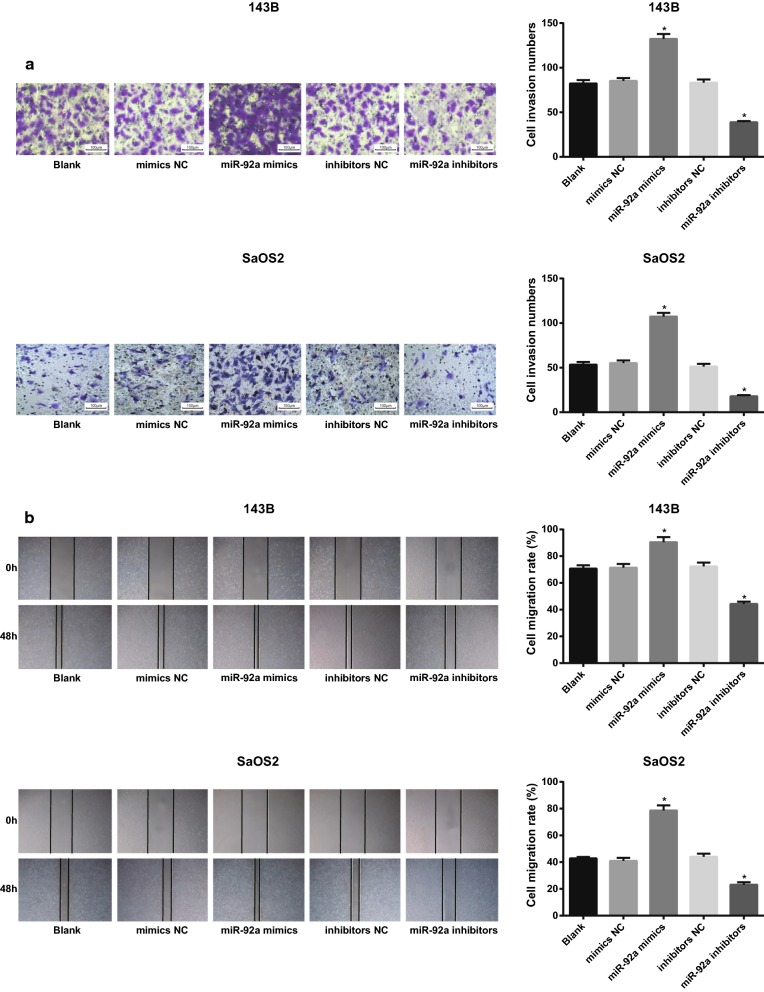
Overexpression of miR-92a promotes invasion and migration of OS cells. **a** Detection of the number of invasive cells in each group by Transwell assay (× 100); **b** detection of cell migration in each group by scratch test; Repetitions = 3; the data was analyzed by one-way ANOVA; After ANOVA analysis, the LSD-t was used for pairwise comparison; *P < 0.05 vs. the blank group

The invasion of 143B and SaOS2 cells in each group was detected by Transwell assay. The results showed that compared with the blank and mimics NC groups, the number of cell invasion was increased significantly in the miR-92a mimics group (both *P* < 0.05). In contrast to the blank and inhibitors NC group, the number of cell invasion in miR-92a inhibitors group was significantly decreased (both *P* < 0.05; Fig. 7a).

The migration ability of 143B and SaOS2 cells in each group was detected by scratch test. The results indicated that the migration rate of cells in the miR-92a mimics group was significantly increased, while that of in the miR-92a inhibitors group was inhibited (both *P* < 0.05; Fig. 7b). These results suggest that miR-92a can promote the invasion and migration of OS cells in vitro, and down-regulating the expression of miR-92a can inhibit the invasion and migration of OS cells in vitro.

### Overexpression of miR-92a promotes growth and metastasis of OS cells in vivo

**Fig. 8:**
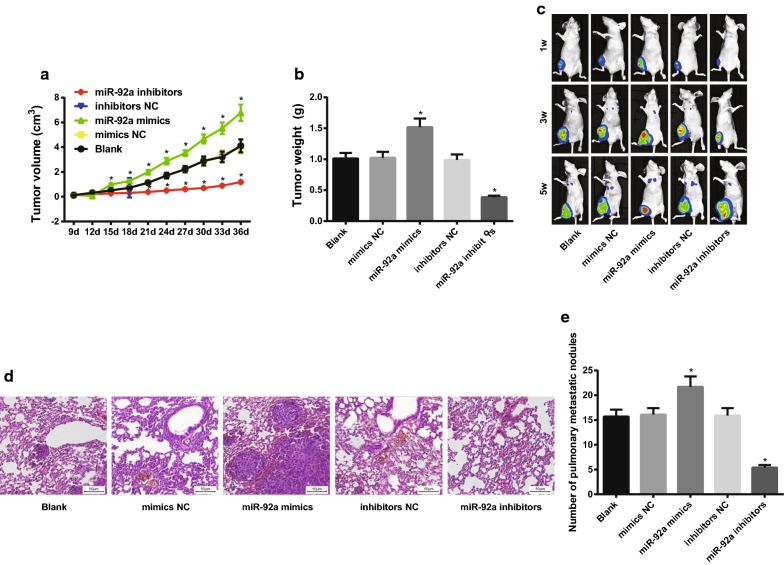
Down-regulation of miR-92a inhibit the growth and metastasis of OS cells in vivo. **a** Tumor growth curve of nude mice in each group; **b** tumor weight of nude mice in each group; **c** lung metastasis of nude mice in each group; **d** observation of lung metastasis in nude mice by HE staining (× 200); **e** the number of lung metastatic nodules in nude mice in each group; N = 6; the data was analyzed by one-way ANOVA; After ANOVA analysis, the LSD-t was used for pairwise comparison; *P < 0.05 vs. the blank group

The results of in situ implantation model of tibia in nude mice showed that compared with the blank group, the tumor growth rate, the tumor volume and tumor weight were increased significantly in the nude mice in the miR-92a mimics group, and the tumor growth rate and tumor weight were significantly decreased in the miR-92a inhibitors group (all *P* < 0.05; Fig. 8a, b).

At 1 week after cell injection, the signal from luciferase bioluminescence was detected in the primary injury of nude mice in each group, and no signal was detected in the lung. At the end of the third week, there was 1 nude mouse with lung metastasis in the blank, mimics NC and inhibitors NC groups. There were 3 nude mice with lung metastasis lesions in the miR-92a mimics group, while there was no lung metastasis in mir-92a inhibitors group. On the 26th and 27th days, only 1 nude mouse was died in the blank and mimics NC group; on the 24th, 29th and 32nd days, there were 3 nude mice died in the miR-92a mimics group, and lung metastasis was confirmed by autopsy. After 32 days, lung metastasis was found in all surviving nude mice in other groups, but only 2 found with lung metastasis in the miR-92a inhibitors group. The results of HE staining showed that the number of lung metastatic nodules in the miR-92a mimics group was significantly higher while the number of lung metastatic nodules in the miR-92a inhibitors group was significantly lower than that in the blank group (Fig. 8c–e). These results suggest that down-regulation of miR-92a can inhibit the growth and metastasis of OS cells in vivo.

### TCF21 is a target gene of miR-92a

**Fig. 9:**
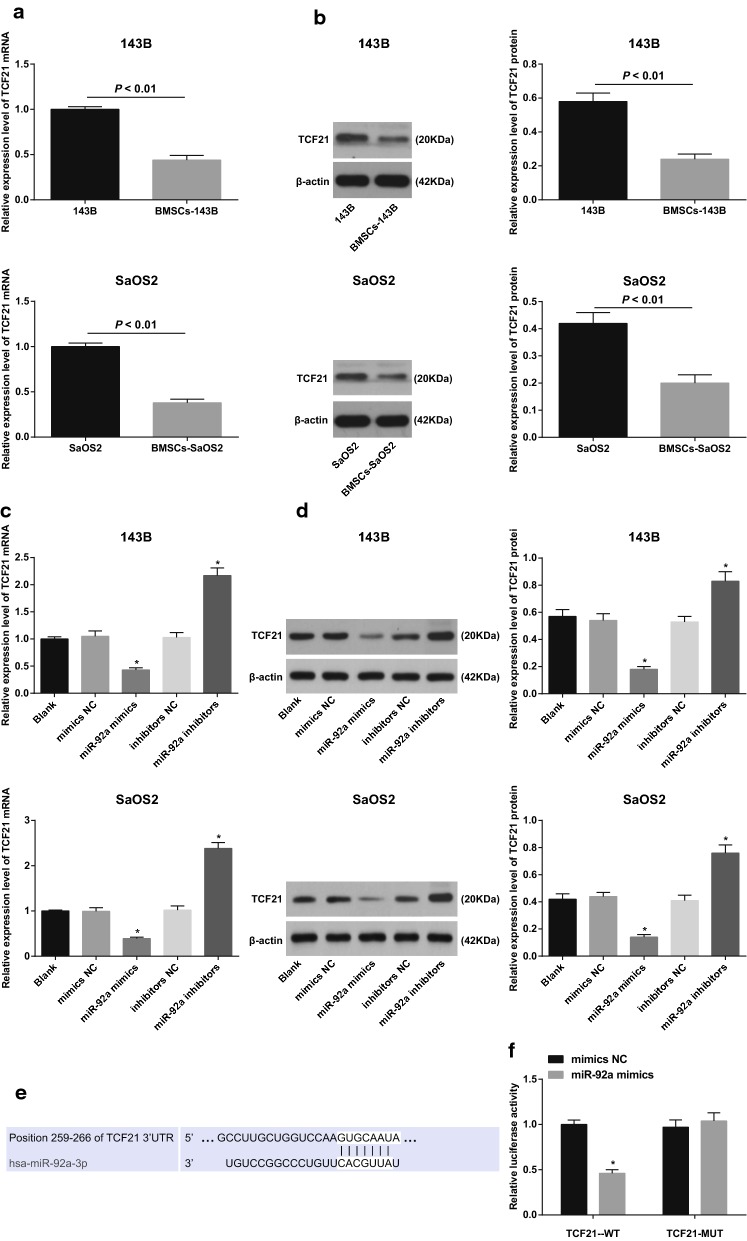
TCF21 is suggested to be a direct target gene of miR-92a. **a** Detection of TCF21 mRNA levels in OS cells before and after co-culture with BMSCs by qRT-PCR; **b** detection of TCF21 protein expression in OS cells before and after co-culture with BMSCs by western blot analysis; **c** detection of TCF21 mRNA expression in OS cells of each group by qRT-PCR; **d** detection of TCF21 protein expression in OS cells by western blot analysis; **e** online prediction of targeting relationship between miR-92a and TCF21; **f** identification of targeting relationship between miR-92a and TCF21 by luciferase activity determination; Repetitions = 3; the data was analyzed by the t test or one-way ANOVA; After ANOVA analysis, the LSD-t was used for pairwise comparison; *P < 0.05 vs. the blank or the mimics NC group

The expression of TCF21 mRNA and protein in 143B and SaOS2 cells were detected by qRT-PCR and western blot analysis before and after co-cultured with BMSCs. The results showed that the mRNA and protein expression of TCF21 were significantly lower in 143B and SaOS2 cells cultured with BMSCs than those in 143B and SaOS2 cells (all *P* < 0.05; Fig. 9a, b), suggesting that TCF21 was involved in the regulation of OS cells by BMSCs.

The mRNA and protein expression of TCF21 in 143B and SaOS2 cells in each group were detected by qRT-PCR and western blot analysis. The results indicated that compared with the blank and mimics NC groups, the expression of TCF21 mRNA and protein in the miR-92a mimics group was significantly decreased (all *P* < 0. 05). Compared with the blank and inhibitors NC groups, the expression of TCF21 mRNA and protein was increased significantly in the miR-92a inhibitors group (all *P *< 0.05; Fig. 9c, d), indicating that miR-92a negatively regulated the expression of TCF21 in OS cells.

Bioinformatics software (http://www.targetscan.org) predicted the targeting relationship between miR-92a and TCF21 (Fig. 9e). The results of luciferase activity determination showed that the relative luciferase activity of TCF21-3′UTR-WT + mimics NC group was significantly lower than that of TCF21-3′UTR-WT + miR-92a mimics group (*P* < 0.05). There was no significant difference in luciferase activity between the TCF21-3′UTR-MUT + mimics NC group and the TCF21-3′UTR-MUT + miR-92a mimics group (*P *> 0.05; Fig. 9f). TCF21 is suggested to be a direct target gene of miR-92a.

### Tissue-based analysis

To verify the results of cells and nude mice experiments, 58 OS specimens were selected and 30 osteochondroma (benign bone lesions) specimens were used as controls. First, ISH was used to detect the expression of miR-92a in OS tissues and osteochondroma tissues. The results showed that 7 of 30 osteochondromas tissues showed positive expression of miR-92a, while 46 of 58 OS tissues showed positive expression of miR-92a. Compared with osteochondroma tissues, the positive expression rate of miR-92a in OS tissues was significantly higher (23.3% VS 79.3%, *P *< 0.01).

**Fig. 10:**
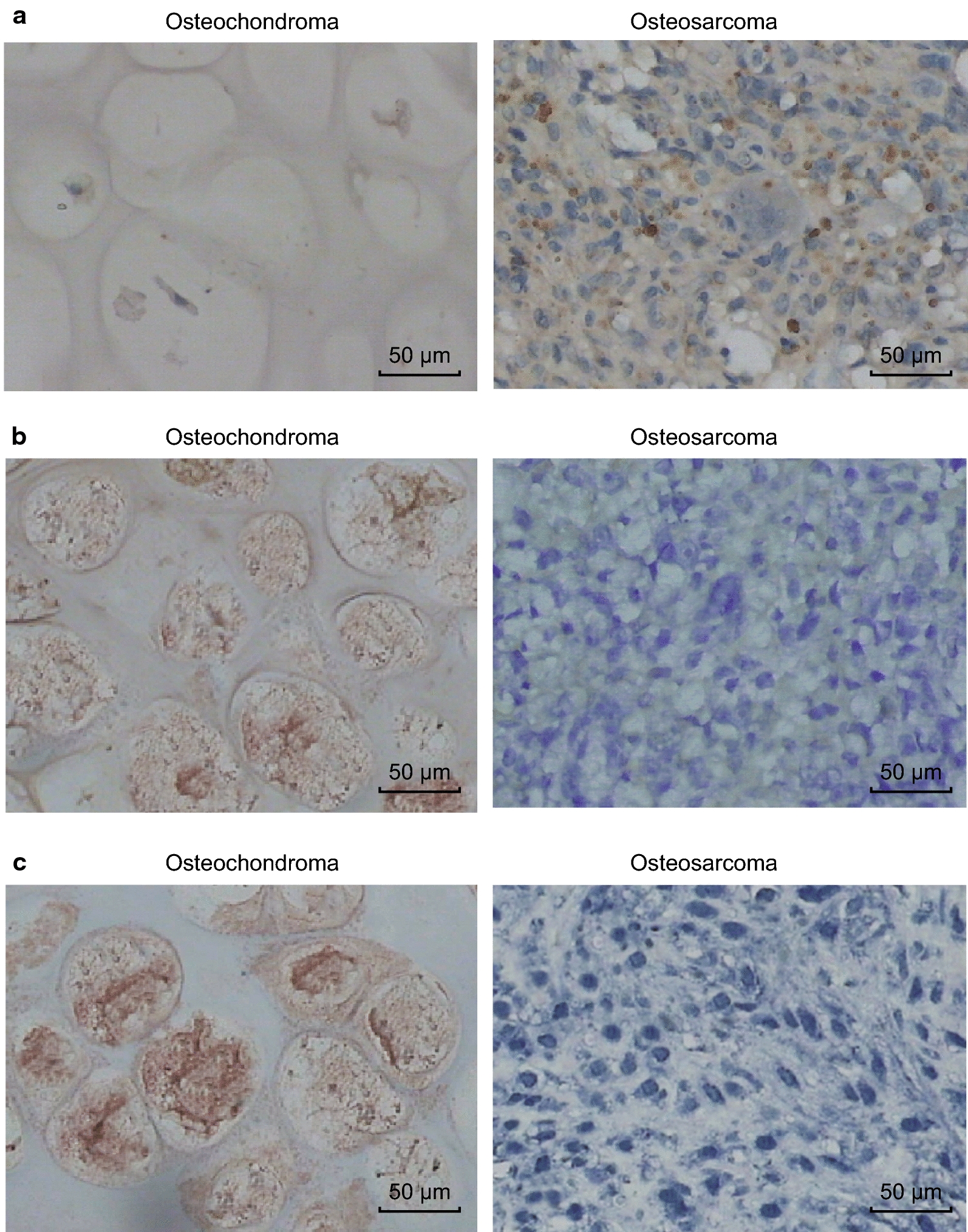
Expression of miR-92a and TCF21 in OS tissues and osteochondroma tissues. **a** In situ hybridization used to detect the expression of miR-92a in tissues (×200); **b** in situ hybridization used to detect the expression of TCF21 in tissues (×200); **c** immunohistochemical detection of TCF21 protein expression in tissues (×200)

Similarly, the expression of TCF21 in OS tissues and osteochondroma tissues was also detected by ISH. The results suggested that 25 of 30 OS tissues showed positive expression of TCF21, while 15 of 58 OS tissues showed positive expression of TCF21. The positive expression rate of TCF21 in OS tissues was significantly lower than that in osteochondroma tissues (25.9% VS 83.3%, *P* < 0.01). In addition, the expression of TCF21 protein in OS tissues and osteochondroma tissues was detected by immunohistochemistry. The results indicated that the positive expression rate of TCF21 protein in OS tissues was significantly lower than that in osteochondroma tissues (29.3% VS 86.7%, *P *< 0.01) (Fig. 10, Table 2).

**Table 2:** Expression of miR-92a and TCF21 in osteochondroma and osteosarcoma tissues [n(%)]

Clinicopathological feature	Osteochondroma (n = 30)	Osteosarcoma (n = 58)	*P*
miR-92a			< 0.01
Positive	7 (23.3)	46 (79.3)	
Negative	23 (76.7)	12 (20.7)	
TCF21 mRNA			< 0.01
Positive	25 (83.3)	15 (25.9)	
Negative	5 (16.7)	43 (74.1)	
TCF21 protein			< 0.01
Positive	26 (86.7)	17 (29.3)	
Negative	4 (13.3)	41 (70.7)	

The relationship between the expression of miR-92a and the clinical characteristics of OS patients was analyzed. The results revealed that in OS tissues, the positive expression rate of miR-92a increased with the increase of Ennecking staging system. The positive expression rate of miR-92a in stage IIB + III patients was significantly higher than that in stage I + IIA patients (*P* < 0.05). The positive expression rate of miR-92a in distant metastatic tumors was significantly higher than that in non-metastatic tumors (*P* < 0.05). However, there was no significant correlation between the expression of miR-92a and age, gender, tumor location and histological type (all *P* > 0.05; Table 3).

**Table 3:** The relationship between the expression of microRNA-92a and clinical characteristics of osteosarcoma patients [n (%)]

Clinicopathological feature	Case	miR-92a expression		*P*
		Positive (n = 46)	Negative (n = 12)	
Age (years)				0.756
≤ 20	31	24 (52.2)	7 (58.3)	
> 20	27	22 (47.8)	5 (41.7)	
Gender				0.342
Male	36	27 (58.7)	9 (75.0)	
Female	22	19 (41.3)	3 (25.0)	
Tumor location				0.603
Femur	22	16 (34.8)	6 (50.0)	
Tibiofibular	20	17 (37.0)	3 (25.0)	
Other	16	13 (28.2)	3 (25.0)	
Histological type				0.107
Osteoblast type	18	11 (23.9)	7 (58.3)	
Chondroblast type	15	12 (26.1)	3 (25.0)	
Fibroblast type	14	13 (28.3)	1 (8.35)	
Mixed type	11	10 (21.7)	1 (8.35)	
Ennecking stage				0.020
I + IIA	21	13 (28.3)	8 (66.7)	
IIB + III	37	33 (71.7)	4 (33.3)	
Distant metastasis				0.009
Yes	30	28 (60.9)	2 (16.7)	
No	28	18 (39.1)	10 (83.3)	

## Discussion

Over the last 3 decades, the introduction of combination chemotherapy has made great advances in the 5-year survival rate of OS patients, while there are many patients respond poorly to chemotherapy and even worse, which have a high susceptibility to local relapse or distant metastasis [22, 23]. In order to improve the outcomes for the treatment of OS patients, it is of great importance to seek for new therapeutics targets the molecular pathway that controls the occurrence and progression of OS cells. Based on this, we conducted this study to investigate the role of miR-92a in metastasis of OS cells in vivo and in vitro by inhibiting expression of TCF21 with the transmission of BMSCs. Collectively, this study demonstrates that BMSCs transfers miR-92a to inhibit expression of TCF21, thereby promoting growth and metastasis of OS in vitro and in vivo.

Initially, the findings in this study suggested that BMSCs promoted proliferation, invasion and migration of OS cells in vitro and promoted the growth and metastasis of OS cells in vivo. As has been reported, BMSCs, are a type of local mesenchymal stem cell, enable to induce breast, prostate, glioblastoma as well as non-small lung cancer cell growth in vivo [24–26]. Besides, the co-culture of breast cancer cells together with mesenchymal stem cells (MSCs) leads to inhibition of tumor growth and reduction of metastasis in vivo [27]. Moreover, BMSCs in mouse has been once used to identify which mesenchymal differentiation properties produce conditions, which enable to favor or inhibit tumorigenesis [28]. All these verified the influence of BMSCs on proliferation and invasion of OS to some extent.

Also, in the present study, high expression of miR-92a was found in OS cells co-cultured with BMSCs. Additionally, overexpression of miR-92a promoted proliferation, invasion and migration of OS cells in vitro and promoted growth and metastasis of OS cells in vivo. There are some studies concentrated on the associations of miR-92a with human cancers. It is suggested that miR-92a is upregulated in non-small cell lung cancer (NSCLC), which could promote growth, metastasis, and chemoresistance of NSCLC cells by targeting phosphatase and tensin homolog (PTEN) [29]. Additionally, miR-92a has also been found to promote pancreatic cancer cell proliferation via the DUSP10/JNK signaling pathway [30]. In prostate cancer, the combination of miR-92a with miR-19b, miR-23b and miR-26a together with the regulation of PTEN and its downstream targets enhance proliferation in prostate cells [31]. Additionally, miR-92a acts as an onco-miRNA in promoting tumor growth of hepatocellular carcinoma (HCC) via inhibition of F-box and WD repeat domain-containing 7 (FBXW7) [32]. Furthermore, miR-92a is involved in the pro-metastasis of nasopharyngeal carcinoma (NPC) by targeting the PTEN/AKT signaling pathway [33]. On the other hand, there are also some studies concentrated on the associations of miR-92a with OS. Gougelet et al. [34] identified miR-92a as a predictive tool in OS, which was upregulated in good responders to ifosfamide. Besides, overexpression of miR-92a was found in OS cell lines [35]. All these aforementioned demonstrates that the expression of miR-92a and its function are implicated in the progression and development of OS, and the mechanism might depend on the involvement of some target genes. In our study, TCF21 was suggested to be a direct target gene of miR-92a. In accordance with the results in our study, TCF21 was significantly downregulated in ovarian cancer tissues, and miR-205 seems to have a pivotal role in the spread of ovarian cancer through binding to TCF21 [36].

Furthermore, for the purpose of verifying the results of cells and nude mice experiments, OS tissues and 30 osteochondroma tissues were selected in our study. The results suggested that compared with osteochondroma tissues, the positive expression rate of miR-92a was significantly higher in OS tissues. Similar to our study, miR-92a was found to be significantly downregulated in OS tissues and cell lines in comparison to adjacent normal tissues and hFOB cells [37]. In addition, the positive expression rate of TCF21 protein in OS tissues was significantly lower than that in osteochondroma tissues. In accordance with the results in our study, a recent study has demonstrated that TCF21 predicted elevated risk of OS, which was related to Enneking stage and potential in forming metastasis of OS [21]. Moreover, the relationship between the expression of miR-92a and the clinical characteristics of OS patients was analyzed. Expression of miR-92a was found to be related to Ennecking staging and distant metastasis in OS patients.

## Conclusion

In summary, our study provides evidence highlighting the role of miR-92a in OS. This study showed that miR-92a upregulation was found in both OS tissues and cell lines. Additionally, miR-92a induced the proliferation and cell cycle progression and inhibited apoptosis of OS cells probably by inhibiting TCF21 expression with the transfer of BMSCs. Altogether, this work supported that miR-92a was considered as a hopeful therapeutic target for the treatment of OS. Future studies are still needed to confirm these findings.
